# The psychological impact of esophageal cancer screening on anxiety and depression in China

**DOI:** 10.3389/fpsyt.2022.933678

**Published:** 2022-10-20

**Authors:** Juan Zhu, Shanrui Ma, Ru Chen, Zhaorui Liu, Zhengkui Liu, Wenqiang Wei

**Affiliations:** ^1^National Central Cancer Registry, National Cancer Center/National Clinical Research Center for Cancer/Cancer Hospital, Chinese Academy of Medical Sciences and Peking Union Medical College, Beijing, China; ^2^Department of Cancer Prevention, The Cancer Hospital of the University of Chinese Academy of Sciences, Zhejiang Cancer Hospital, Institute of Cancer and Basic Medicine, Chinese Academy of Sciences, Hangzhou, China; ^3^Institute of Mental Health, Key Laboratory of Mental Health, Ministry of Health, Peking University, Beijing, China; ^4^Chinese Academy of Sciences, Key Laboratory of Mental Health, Institute of Psychology, Beijing, China

**Keywords:** esophageal cancer, screening, endoscopy, anxiety, depression, psychological impact, high-risk regions

## Abstract

**Objective:**

The psychological impact of screening is unclear and has been ignored. This study aimed to evaluate the psychological impact of esophageal cancer (EC) screening on anxiety and depression in China.

**Materials and methods:**

A multicenter, population-based study in five high-risk regions of EC was conducted from 2019 to 2020. Residents were recruited and underwent endoscopic screening and then were diagnosed with normal, esophagitis, low-grade intraepithelial neoplasia (LGIN), high-grade intraepithelial neoplasia (HGIN) and EC. Subjects who did not participate in the screening were referred to as the control group. We surveyed their anxiety and depression levels at baseline and after endoscopy and informed them of different pathological results to evaluate the psychological impact of the screening process.

**Results:**

A total of 2,337 subjects completed all surveys in the screening process (normal: 355, esophagitis: 1,713, LGIN: 213, HGIN: 43 and EC: 13), with 63 controls. The levels of anxiety and depression of screeners were significantly higher than those of controls (*P* < 0.001). The fluctuation of anxiety and depression showed a “V” pattern in the screening process. The prevalence of anxiety symptoms at baseline, after endoscopy and after knowing the pathological results was 5.6, 0.3, and 3.2%, respectively (*P* < 0.001), and the corresponding prevalence of depression was 3.6, 0.2, and 2.1%, respectively (*P* < 0.001). With the aggravation of pathological results, the levels of anxiety and depression increased significantly (*P* < 0.001), especially in patients informed of HGIN (16.3 and 9.3%) and EC (23.1 and 30.8%).

**Conclusion:**

Participation in endoscopic screening may bring short-term adverse psychological effects, especially at baseline and knowing the pathological results. More attention should be given to participants waiting for endoscopic screening. The method of informing the screening results of HGIN and EC should be improved. Further precise screening is needed to concentrate on high-risk groups to reduce the psychological impact of screening.

## Introduction

Cancer screening is a double-edged sword (1). Although strong evidence from large population-based studies has confirmed the effectiveness of endoscopic screening in reducing EC incidence and mortality (2–6), the psychological impact of cancer screening may question the overall benefit of endoscopic screening, such as increased psychological distress due to the positive results of the screening. A population-based screening project aimed to ensure that the benefits outweigh potential harm, including psychological impact (7). The US Preventive Services Task Force (USPSTF) suggested that psychological effects should be considered in the net effectiveness of cancer screening (8). The current national cancer screening recommendations also mention the psychological disorders caused by cancer screening (8).

Some preliminary evidence supports the association between cancer screening and negative emotions (9). Among those who received abnormal screening results, the potential psychological harm caused by screening was particularly obvious (10). The UK lung cancer screening trial reported that participants with abnormal screening results had higher psychological pain in the short term than those with negative screening results (11). For endoscopic screening of EC, waiting for an invasive endoscopy examination may trigger or increase participants’ anxiety and depression symptoms. In addition, the worry about the screening results and positive diagnosis may increase their psychological distress. The probability of screening benign diseases is high (such as esophagitis and LGIN). The progression of such a disease is slow and generally does not affect patient survival. These slightly abnormal results may lead to overdiagnosis and unnecessary anxiety and depression to some extent, and patients have been living under the yoke of screening results (12). Participants screened and diagnosed with precancerous lesions and cancer (HGIN and EC) had a higher risk for cancer and recurrence and may be more likely to have psychological pressure and negative psychological emotions (13, 14).

The potential psychological outcomes of cancer screening are largely underestimated (15). Previous studies on the psychological impact of cancer screening are inconsistent. Current research in this field mainly focuses on the following types of cancer: lung cancer, breast cancer, colorectal cancer, cervical cancer and ovarian cancer (7, 16–18). However, research on the psychological impact caused by EC screening is still limited and unclear. Therefore, a multicenter population-based study was conducted in high-risk regions of China to evaluate the psychological impact of EC screening on anxiety and depression in China, including baseline, after endoscopy and informing screening diagnosis.

## Materials and methods

### Study design and participants

**FIGURE 1:**
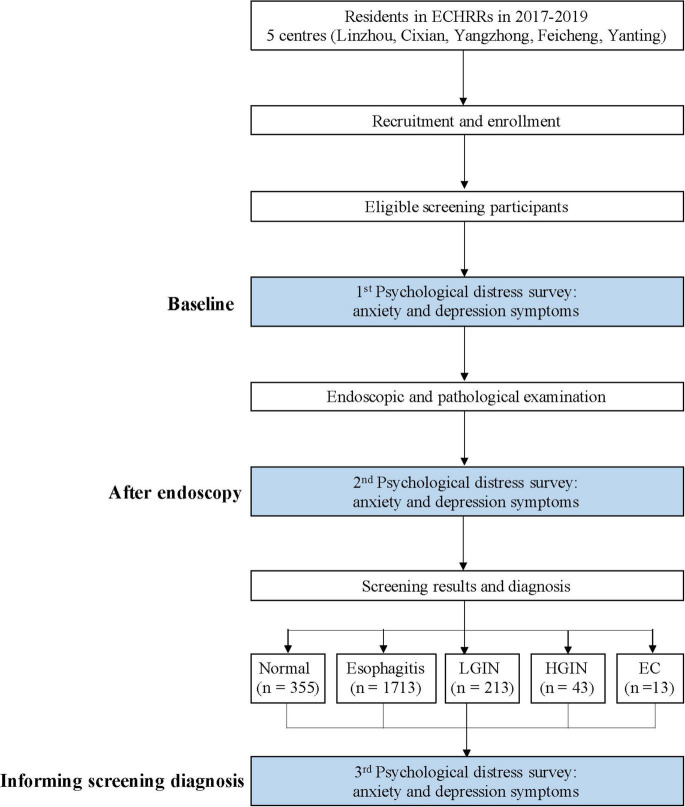
Flowchart of the study.

Based on the National Cohort of Esophageal Cancer (NCEC), we carried out a multicenter population-based survey in 5 areas of China where the risk of EC is high (Linzhou, Cixian, Yangzhong, Feicheng, and Yanting) from 2019 to 2020. Permanent residents aged 40–60 years were the target subjects. During the enrollment period, those residents were informed through various formats, such as broadcasting and brochures. They were invited by local well-trained investigators. According to the inclusion criteria, individuals who had no emergency symptoms and no history of cancer and who were mentally and physically competent were enrolled for endoscopic screening after signing informed consent forms. Residents underwent endoscopic screening and were then diagnosed with normal, esophagitis, low-grade intraepithelial neoplasia (LGIN), high-grade intraepithelial neoplasia (HGIN) and EC. Subjects who did not participate in the screening were referred to as the control group. We surveyed their anxiety and depression levels at baseline (waiting for endoscopic screening), after endoscopy (no more than 24 h after endoscopy) and informed them of different pathological results (within 1 week after knowing the screening results) to evaluate the psychological impact on the screening process. The study flowchart is shown in Figure 1.

### Measurement

#### Anxiety symptoms

Anxiety symptoms were evaluated using the Chinese version of the seven-item Generalized Anxiety Disorder (GAD-7), a measurement tool widely used and acknowledged around the world. Previous studies have shown the tool’s good reliability and validity in primary medical care and clinical practice (19). The GAD-7 is used to identify anxiety symptoms of individuals in the past 2 weeks, with 7 items and 4 response scores representing the frequency of each item (0 representing never; 1 representing sometimes; 2 representing more than half of the time; 3 representing almost every day). The anxiety score is calculated by adding the answers for each of the items and ranges from 0 to 21. The higher the score is, the worse the anxiety symptoms are. The prevalence of depression was assessed using the GAD-7 scoring algorithm. A score of 5 was regarded as the threshold for positive anxiety symptoms (20).

#### Depression symptoms

The nine-item Patient Health Questionnaire (PHQ-9), one of the most well-known tools for assessing depression symptoms (21), is valid and reliable for assessing current depressive disorder (22, 23). The PHQ-9 is a self-report questionnaire composed of 9 items that correspond to the Statistical Manual of Mental Disorders-IV (DSM-IV) diagnostic criteria for major depressive episodes. The recall period corresponds to the previous 2 weeks, and the response scale ranges from 0 (not at all) to 3 (nearly every day). The PHQ-9 score is the sum of all items. The higher the score is, the worse depression symptoms are. Based on results from a previous validation study, for the current study, we considered a PHQ-9 score of 5 or higher to indicate the presence of depression symptoms (23).

### Quality control

The overall study protocol, standard operational procedure, and instructions for interviews were formulated by an expert panel. The screening outcome of participants was diagnosed by endoscopists and pathologists. All endoscopic examinations were conducted by well-trained endoscopists at local hospitals according to the guidelines for EC screening and early diagnosis and treatment in China. Two pathologists independently read the biopsy slides, and any disagreements were resolved through discussion with a third pathologist. The psychological outcomes of participants were evaluated by well-trained investigators. We conducted a series of training programs for the investigators, which included the study protocol, an introduction to the questionnaire, interview procedures, and communication skills. The investigators ensured that the target subjects were notified by telephone or by visits. Face-to-face or self-administered interviews were conducted or managed by well-trained local interviewers to ensure that the questionnaire was fully completed. Ethical approval for the study was received in China from the Ethics Committee of the Cancer Institute and Hospital, Chinese Academy of Medical Sciences (Approval number 16-171/1250).

### Statistical analysis

The variance trend test for the score was used to compare the mean score of anxiety and depression among three timing points (baseline, after endoscopy and knowing screening diagnosis. The χ^2^ trend test was applied to compare the positive prevalence of anxiety and depression at the three time points. Analysis of variance and χ^2^ test were used to compare the levels of anxiety and depression among different pathological grades. Bonferroni adjustment was used to compare the levels of anxiety or depression between the two groups. The same superscript letter indicates no significant difference, and different letters indicate a significant difference. Data management, programming, and analyses were carried out using SAS 9.4 (SAS Institute Inc., Cary, NC, USA). Two-sided *P* < 0.05 was considered statistically significant.

## Results

### Baseline characteristics of the screened participants

A total of 2,337 subjects completed all surveys in the screening process (normal: 355, esophagitis: 1,713, LGIN: 213, HGIN: 43 and EC: 13), with 63 controls. The mean age of the screeners was 58.3 ± 6.4 years old. Most participants were educated in high school or below (99.3%). The majority of household income was 3.0–7.0 ten thousand RMB (36.9%). 78.3 and 69.0% of screeners had no history of smoking and alcohol consumption, respectively, and 16.2% of respondents rated their health as just so-so (Supplementary Table 1).

### Anxiety and depression in the screening process

**FIGURE 2:**
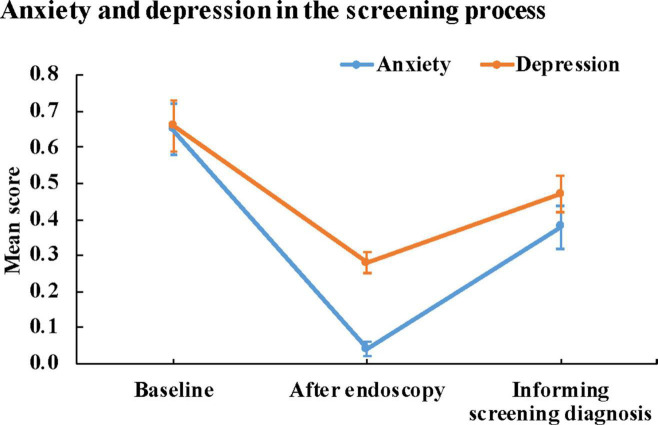
Anxiety and depression in the screening process.

As shown in Figure 2 and Table 1, the levels of anxiety and depression of screeners were significantly higher than those of controls (*P* < 0.001). The mean score of anxiety and depression in the control groups were 0.11 and 0.17, respectively, and the corresponding prevalence of anxiety and depression were 1.6 and 1.6%, respectively.

**TABLE 1:** Anxiety and depression in the screening process.

	Group and timing	Mean (95% CI)	n (Prevalence,%)	P for trend*	*P for trend^#^*
**Anxiety**	Control	0.11 (−0.11–0.33)^b^	1 (1.6)^a,b^		
	Screening				
	Baseline	0.65 (0.58–0.72)^a^	130 (5.6)^b^		
	After endoscopy	0.04 (0.02–0.06)^b^	8 (0.3)^b^	<0.001	<0.001
	Informing screening diagnosis	0.38 (0.32–0.44)^c^	74 (3.2)^a^		

**Depression**	Control	0.17 (−0.04–0.38)^b^	1 (1.6)^a,b^		
	Screening				
	Baseline	0.66 (0.59–0.72)^a^	84 (3.6)^b^		
	After endoscopy	0.28 (0.25–0.31)^b^	5 (0.2)^b^	<0.001	<0.001
	Informing screening diagnosis	0.47 (0.42–0.53)^c^	49 (2.1)^a^		

n: frequency of anxiety/depression symptoms. *Variance trend test for score; ^#^Chi-square trend test for prevalence; Bonferroni adjustment was used to compare the levels of anxiety or depression between the two groups. The superscript containing the same letter indicates no statistical difference, and different letters indicate statistical difference.

The fluctuation of anxiety and depression showed a “V” pattern in the screening process. The mean score (95% CI) of anxiety at baseline, after endoscopy and knowing screening results were 0.65 (0.58–0.72), 0.04 (0.02–0.06) and 0.38 (0.32–0.44) (*P* < 0.001), and the corresponding score (95% CI) of depression in the screening process were 0.66 (0.59–0.72), 0.28 (0.25–0.31) and 0.47 (0.42–0.53), respectively (*P* < 0.001). The prevalence of anxiety symptoms at baseline, after endoscopy and after knowing the pathological results was 5.6, 0.3, and 3.2%, respectively (*P* < 0.001), and the corresponding prevalence of depression in the screening process was 3.6, 0.2, and 2.1%, respectively (*P* < 0.001). The levels of anxiety and depression among screeners were high at baseline and after knowing the pathological diagnosis (all *P* < 0.001).

### Anxiety and depression in the screening process, by pathological grade

**FIGURE 3:**
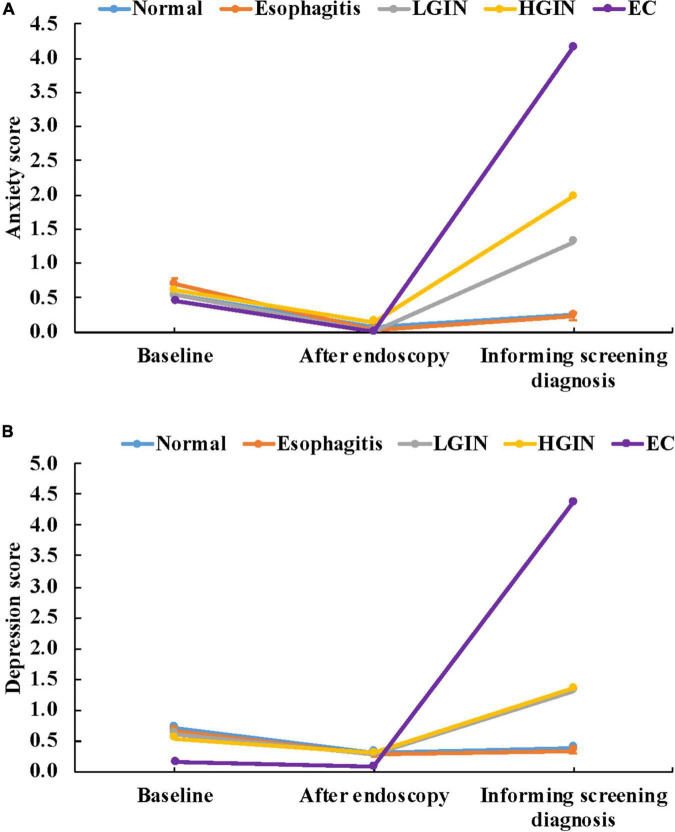
**(A)** Anxiety in the screening process by pathological grade. **(B)** Depression in the screening process by pathological grade.

As shown in Figure 3 and Table 2, the levels of anxiety and depression also showed a “V” pattern in the screening process, which were high at baseline and after knowing the pathological diagnosis (all *P* < 0.001). The mean score of anxiety for subjects who screened for LGIN at baseline, after endoscopy and after informing the screening diagnosis were 0.54, 0.01, and 1.31, respectively (*P* < 0.001). The mean score of anxiety for subjects diagnosed with HGIN in the screening process was 0.60, 0.14, and 1.98 (*P* < 0.001), and the corresponding score of anxiety for EC patients in the screening process was 0.46, 0.00, and 4.15 (*P* = 0.024). Similar patterns related to depression were shown in the screening process.

**TABLE 2:** Anxiety and depression in the screening process, by pathological grade.

		Anxiety			Depression		
		Baseline	After endoscopy	Informing screening diagnosis	Baseline	After endoscopy	Informing screening diagnosis
**Normal**							
	Mean score	0.54	0.07	0.25^a^	0.71	0.31	0.39^a^
	n (Prevalence,%)	17 (4.8)	3 (0.9)	7 (2.0)	21 (5.9)	2 (0.6)	5 (1.4)
	P for trend*	0.001			<0.001		
	*P for trend^#^*	0.017			<0.001		
**Esophagitis**							
	Mean score	0.69	0.03	0.23^a^	0.66	0.27	0.34^a^
	n (Prevalence,%)	99 (5.8)	4 (0.2)	27 (1.6)	54 (3.2)	3 (0.2)	9 (0.5)
	P for trend*	<0.001			<0.001		
	*P for trend^#^*	<0.001			<0.001		
**LGIN**							
	Mean score	0.54	0.01	1.31^b^	0.61	0.29	1.31^b^
	n (Prevalence,%)	11 (5.2)	0 (0.0)	30 (14.1)	9 (4.2)	0 (0.0)	27 (12.7)
	P for trend*	<0.001			<0.001		
	*P for trend^#^*	<0.001			<0.001		
**HGIN**							
	Mean score	0.60	0.14	1.98^c^	0.53	0.30	1.35^b^
	n (Prevalence,%)	2 (4.7)	1 (2.3)	7 (16.3)	0 (0.0)	0 (0.0)	4 (9.3)
	P for trend*	0.011			0.014		
	*P for trend^#^*	0.045			0.022		
**EC**							
	Mean score	0.46	0.00	4.15^d^	0.15	0.08	4.38^c^
	n (Prevalence,%)	1 (7.7)	0 (0.0)	3 (23.1)	0 (0.0)	0 (0.0)	4 (30.8)
	P for trend*	0.024			0.002		
	*P for trend^#^*	0.202			0.017		
*P1*		0.463	0.245	<0.001	0.693	0.689	<0.001
*P2*		0.938	0.066	<0.001	0.066	0.091	<0.001

*Variance trend test for score; ^#^Chi square trend test for prevalence; P1: Analysis of variance; P2: Chi-square test; Bonferroni adjustment was used to compare the levels of anxiety or depression between the two groups. The superscript containing the same letter indicates no statistical difference, and different letters indicate statistical difference.

There was no significant difference in anxiety and depression among each pathological grade at baseline (*P* = 0.463) and after endoscopy (*P* = 0.245). Different pathological results lead to the diversity of their anxiety and depression (*P* < 0.001). The prevalence of anxiety when informing normal, esophagitis, LGIN, HGIN and EC were 2.0, 1.6, 14.1, 16.3, and 23.1%, and the corresponding prevalence of depression for each pathological grade were 1.4, 0.5, 12.7, 9.3, and 30.8%. With the aggravation of pathological results, the levels of anxiety and depression increased significantly (*P* = 0.011), especially in patients informed of LGIN, HGIN, and EC.

## Discussion

To our knowledge, this is the first multicenter, multi-stage study to estimate psychological impact of EC screening at home and abroad. The findings supported that participating in endoscopic screening for EC may have adverse psychological effects (increased anxiety and depression levels). The fluctuation of anxiety and depression showed a “V” pattern in the screening process. Anxiety and depression were obvious at baseline and when the pathological results were known. With the aggravation of pathological results, the levels of anxiety and depression increased significantly, especially in patients informed of HGIN and EC. Our findings gave us a better understanding of evaluating the effectiveness of EC screening and provided informative evidence for optimization of the screening process. Proper psychological consultation and intervention should be provided to those waiting for EC screening. And it is necessary to improve the way of informing the pathological results of HGIN and EC. In addition, further precise screening is needed to concentrate on high-risk groups to reduce the psychological impact of screening. The findings provided scientific evidences to cancer screening research, and were informative for screening strategy.

In the study, compared with controls, residents who participated in endoscopic screening may have short-term adverse psychological effects. A previous study by our team indicated that the health-related quality of life and utility of people who underwent breast cancer screening were lower than those of the general population, and anxiety and depression were the most prominent dimensions (24). The main reasons for increased anxiety and depression at baseline are as follows: (a) Screeners actively participated in the screening and generally paid more attention to their health; (b) Most of the screened people were middle-aged and elderly people in rural areas, with a relatively low educational level and poor health literacy on EC. The phenomenon of “cancer phobia” is common in high-risk regions in China. Residents lacked a correct understanding of electronic gastroscopy, and felt fear and timidity of the invasiveness of endoscopy. The psychological problems caused by endoscopic screening were obvious because the process was invasive, which challenged their psychological status and increased the level of anxiety and depression symptoms, and they may even give up halfway. Several studies indicate that the acceptance and participation rate of upper gastrointestinal endoscopy was low because the invasive examination process was very uncomfortable (25, 26).

The fluctuation of anxiety and depression showed a “V” pattern in the screening process. The anxiety and depression were obvious at baseline and when the pathological results were known and relieved after endoscopy. A wide range of studies have consistently proposed that anxiety and depression may play vital and unfavorable roles in increasing the risk of cancer incidence and mortality and in worsening prognosis (27–30). At present, few studies have reported the psychological effects of EC screening. Related research at home and abroad on the psychological impact of screening mainly focuses on lung cancer, breast cancer, colorectal cancer and cervical cancer. We summarized evidence on the psychological impact of cancer screening and found that related evidence is controversial. (a) Low-dose spiral CT screening for lung cancer: A previous study has pointed out that the score of anxiety and depression increased significantly after diagnosis (31). Several studies have also shown that the fear of cancer and psychological distress of participants who received uncertain nodules or abnormal screening results increased obviously in a short-term period, but the difference may be not clinically significant (7, 11, 32). In Contrast, several studies have pointed out that the diagnosis of pulmonary nodules by lung cancer screening does not seem to lead to adverse psychological harm (18, 33); (b) Breast cancer screening: 70% of participants with positive screening results of breast cancer suffered from severe psychological distress and increased anxiety and depression, especially for patients whose screening results need further examination. No psychological impact was found in those who received negative screening results (17). (c) Colorectal cancer screening: Previous research has indicated positive results of fecal immunochemical tests and subsequent coloscopy hurt screeners (34). Other studies supported that participating in the screening of colorectal cancer cannot have any negative psychological impacts on the subjects. There was no psychological difference between those who detected polyps and those who received negative results (35, 36). (d) Cervical cancer: Several studies have shown that an abnormal diagnosis of cervical cancer caused negative emotions, such as increased anxiety levels (16, 37). (e) Gastrointestinal cancer: A prior study in Iran found that informing the results of screening may lead to a psychological burden for screeners (38). The levels of anxiety and depression of individuals who knew the diagnosis were significantly higher than those who did not know the results.

In addition to the above controversy on the psychological impact of cancer screening, there is another voice that screening and diagnosis may have positive psychological effects. Although the screening results may not be reassuring, screening tools diagnosed with early stage lung cancer or early stage pancreatic cancer felt comfortable and assured because they had not developed to the late stage and had access to comprehensive treatment. Early treatment could improve their prognosis and quality of life (39, 40). It is considered wise and lucky to participate in colorectal cancer screening because of the early detection of cancer (41). Another study pointed out that prostate cancer screening brought reassurance effects for men (42). In addition, the normal results of ovarian cancer screening may have positive psychological effects, including positive emotions and increased belief in the effectiveness of screening (43). For the above three contradictory findings (negative psychological impact, no psychological impact and positive psychological effects), the possible explanation is that the measurement tools and methods used in these studies are different, and the social and cultural backgrounds of the objects are diverse.

In this study, we found that the levels of anxiety and depression increased significantly with the aggravation of pathological results, especially in patients informed of HGIN and EC. A possible explanation is that esophagitis is mild and low risk and can even be considered a subhealth status, and no clinical treatment is needed. Those patients were relieved and relaxed after knowing the pathological results. When informed of LGIN, HGIN, and EC, those patients need clinical treatment, such as mucosal resection. They are worried about disease progression and metastasis, suffer a heavy psychological burden due to uncertainty, and live in the shadow of precancerous lesions and EC. A cancer-related diagnosis at screening may act as a serious stressor and stimulate stressful life events, especially for patients screened as having HGIN or EC. These patients are susceptible to falling into a gloomy, depressed and painful mood, which is detrimental to disease prognosis by its effect on neuroendocrine-immune function through the hypothalamic–pituitary–adrenal axis (HPA) (44–46). For HGIN, the risk of EC is nearly 28 times higher than that of normal people (47). Previous research indicated that the method of informing positive results of screening played a vital role in psychological outcomes (11). The findings suggested that the method of informing abnormal diagnosis of screening should be improved. Targeted psychological interventions can be added when issuing pathetical reports. Doctors fully inform the meaning of the screening results and the benefits of screening, such as early diagnosis and early treatment to improve prognosis, to reduce excessive concerns about the screening results. We can also establish a network system to monitor the psychological changes of screeners.

To our knowledge, this is the first population-based, multicenter study to evaluate the potential psychological effects of endoscopic screening for EC. Based on its endoscopic screening protocol in multiple areas of China with a high incidence of esophageal cancer, key timing points in the screening were investigated, including baseline, endoscopy and informing screening results. The design is innovative and convincing to some extent. The third advantage is that both anxiety and depression symptoms, which are major manifestations of psychological distress, were all measured by validated instruments. The GAD-7 and PHQ-9 have emerged as powerful tools for studying anxiety and depression disorders. In addition, the consistent results of anxiety and depression symptoms make the findings more objective and credible.

Some limitations of our work should be acknowledged. First, the samples of controls were relatively small, the difference between screening group and control group may need to be interpreted with caution. We will expand the sample size to validate the results in the future. Second, anxiety and depression symptoms were evaluated in the study, which was not a clinical diagnosis for anxiety and depression disorders. In the future, psychiatrists should be involved in the screening process, and more objective indicators could be considered. Third, the cutoff value of 5 for the prevalence of anxiety and depression may overestimate the results. In addition, subjects in the study were those who actively participated in the screening and paid more attention to their health. Volunteer bias may exist in the study, and the results may not be generalizable to the general population. Although we tried to adjust for related confounding factors as much as possible to offset and reduce confounding effects, unmeasured and residual confounding may interfere with the interpretation and validity of the results. For example, subjective data may cause residual confounding. Although multi-round training was conducted for investigators, inevitably bias still exists.

## Conclusion

In summary, participation in endoscopic screening may bring short-term adverse psychological effects. The fluctuation of anxiety and depression showed a “V” pattern in the screening process. Anxiety and depression were obvious at baseline and when the pathological results were known, especially for those who were told they had HGIN or esophageal cancer. The findings provide useful suggestions for optimizing the screening process. More attention should be given to participants at baseline. The method of informing the screening results of HGIN and EC should be improved. The negative psychological impact of screening should be considered when comprehensively evaluating the effectiveness of cancer screening. The results suggested that it was necessary to further explore methods of concentrating high-risk groups, reducing the number of endoscopic screening participants, and improving the esophageal cancer screening detection rate to reduce the psychological impact of screening.

## Data availability statement

The original contributions presented in the study are included in the article/Supplementary material, further inquiries can be directed to the corresponding author.

## Ethics statement

The studies involving human participants were reviewed and approved by the National Cancer Center/National Clinical Research Center for Cancer/Cancer Hospital, Chinese Academy of Medical Sciences and Peking Union Medical College (No. 16-171/1250). The patients/participants provided their written informed consent to participate in this study.

## Author contributions

JZ: conceptualization, methodology, investigation, data curation, and writing – original draft. SM: data curation and writing – reviewing. RC, ZL, and ZKL: conceptualization. WW: conceptualization, supervision, project administration, and funding acquisition. All authors contributed to the article and approved the submitted version.

## Abbreviations

EC: esophageal cancer
GAD-7: seven-item generalized anxiety disorders
HGIN: high-grade intraepithelial neoplasia
LGIN: low-grade intraepithelial neoplasia
PHQ-9: nine-item patient health questionnaire.
