# Supplementary material for: The psychological impact of esophageal cancer screening on anxiety and depression in China
Source: Front Psychiatry. 2022 Oct 20;13:933678. doi: 10.3389/fpsyt.2022.933678 (PMC9630588; doi:10.3389/fpsyt.2022.933678)
Supplement: Supplementary file 1 [file Table_1.DOCX]

**TABLE S1 |** Baseline characteristics of the screening participants

| **Variables** | | **Frequency (n)** | **Percentage (%)** |
| --- | --- | --- | --- |
| Total Sample | | 2337 | 100.0 |
| Age, years (Mean ± SD) | | 58.3 ± 6.4 | |
| High risk regions | |  |  |
|  | East: Cixian | 601 | 25.7 |
|  | East: Yangzhong | 972 | 41.6 |
|  | East: Feicheng | 276 | 11.8 |
|  | Central: Linzhou | 354 | 15.2 |
|  | West: Yanting | 134 | 5.7 |
| Gender | |  |  |
|  | Male | 1016 | 43.5 |
|  | Female | 1321 | 56.5 |
| Marital status^*^ | |  |  |
|  | Married | 153 | 6.6 |
|  | Others | 2184 | 93.5 |
| Highest education level | |  |  |
|  | Primary school or below | 951 | 40.7 |
|  | Junior or Senior high school | 1369 | 58.6 |
|  | Undergraduate or above | 17 | 0.7 |
| Household income, 10,000RMB | |  |  |
|  | <3.0 | 330 | 14.1 |
|  | 3.0-7.0 | 863 | 36.9 |
|  | 7.0-11.0 | 673 | 28.8 |
|  | ≥11.0 | 471 | 20.2 |
| Smoking | |  |  |
|  | Never | 1829 | 78.3 |
|  | Sometimes | 51 | 2.2 |
|  | Regular | 456 | 19.5 |
| Alcohol drinking | |  |  |
|  | Never | 1613 | 69 |
|  | Sometimes | 335 | 14.3 |
|  | Regular | 389 | 16.7 |
| Self-rated health | |  |  |
|  | Good | 1958 | 83.8 |
|  | Just so so | 379 | 16.2 |

^*^ Others: including the unmarried, divorced or widowed
